# Experimental data on the photoelectrochemical oxidation of phenol: Analysis of pH, potential and initial concentration

**DOI:** 10.1016/j.dib.2019.103949

**Published:** 2019-04-25

**Authors:** J.A. Villota-Zuleta, J.W. Rodríguez-Acosta, S.F. Castilla-Acevedo, N. Marriaga-Cabrales, F. Machuca-Martínez

**Affiliations:** Escuela de Ingeniería Química, Universidad del Valle, GAOX, Santiago de Cali, Valle Del Cauca, 76001, Colombia

**Keywords:** Photocurrent, Supporting electrolyte, Energy consumption, Kinetic performance, Chronoamperometry, Linear voltammetry

## Abstract

The data collected in the present work correspond to percentages of phenol degradation by means of photoelectrochemical oxidation (PEC). Also, the information related to the energetic and kinetic performance of this advanced oxidation process (AOPs) is shown. The tests were divided into two stages: 1. Supporting electrolytes tests to determine the electrolyte that presents a better response to photocurrent and 2. Degradation of phenol to obtain the adequate conditions for the elimination of the contaminant. A central rotary composite design with uniform precision at two levels was used to analyze the influence of the initial pH, electrode potential and the initial concentration of substrate. Finally, with all the data obtained, calculation of degradation rates and the electrical energy per order EEO were made.

**Table:** Specifications table

Subject area	*Chemical Engineering, environmental engineering, water treatment.*
More specific subject area	*Electrochemistry, Advanced oxidation processes*
Type of data	*Figure and table*
How data was acquired	*Linear voltammetry, chronoamperometry and direct photometric method described in the ASTM D1783 standard for phenol analysis. Potentiostat/Galvanostat Serie G750, Gamry. UV-VIS 1800 Spectrophotometer, Shimadzu.*
Data format	*Analyzed*
Experimental factors	*All experimental test was performed to laboratory-scale in a three-electrode cell. Four UV-A LEDs (3W,* 25 mW/cm^2^*) were used as illumination source. A central rotary composite design with uniform precision at two levels was used.*
Experimental features	*The experimental data were obtained to evaluate the effect of the initial pH, electrode potential and initial concentration of substrate on the photoelectrochemical oxidation of phenol. In addition, it was evaluated the synergy effects of the photolysis, photocatalysis and anodic oxidation on the global process.*
Data source location	*GAOX Group, Chemical Engineering School, Advanced oxidation processes laboratory, Universidad del Valle, Cali, Colombia.*
Data accessibility	*The data are available only in this article*
Related research article	*H. Zhang, H. Ding, X. Wang, C. Zeng, A. Lu, Y. Li, & C. Wang. Photoelectrochemical performance of birnessite films and photoelectrocatalytic activity toward oxidation of phenol. Journal of Environmental Sciences 52 (2017) 259–267.*http://doi.org/10.1016/j.jes.2016.04.009

**Table:** 

**Value of the data** • Data can be used to compare different advanced oxidation processes using performance standard indicators. • The data obtained could be used to check the effect of supporting electrolyte in the photoelectrochemical oxidation process. • The data could be also useful for scaling up and economic analysis for wastewater treatment. • The raw data are presented in this data article allow to do analysis with the raw data sets.

## 1 Data

### 1.1 Supporting electrolytes tests

**Fig. 1:**
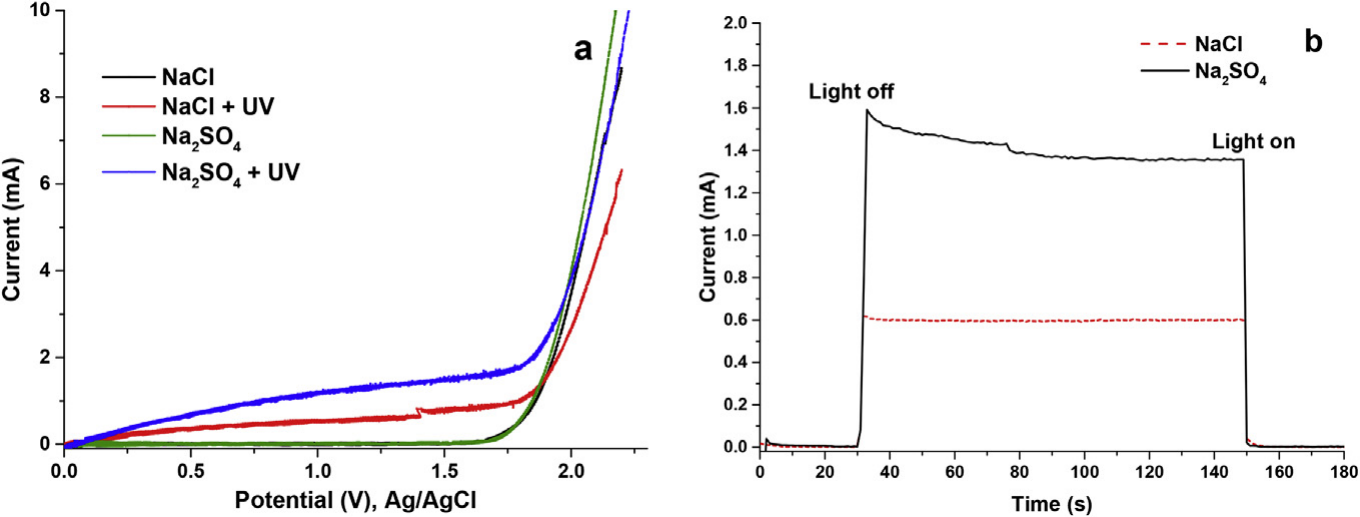
Voltammetry and Chronoamperometry of the electrolytes. a) Linear scanning voltammetry b) Chronoamperometry.

Fig. 1a and Fig. 1b show a voltammogram and a chronoamperometry of 0.1M of ${\text{Na}}_{2}{\text{SO}}_{4}$ and NaCl as solution electrolytes in the absence and presence of light.

All the data reported for the following tests were obtained using $Na_{2}SO_{4}$ as supporting electrolyte.

**Fig. 2:**
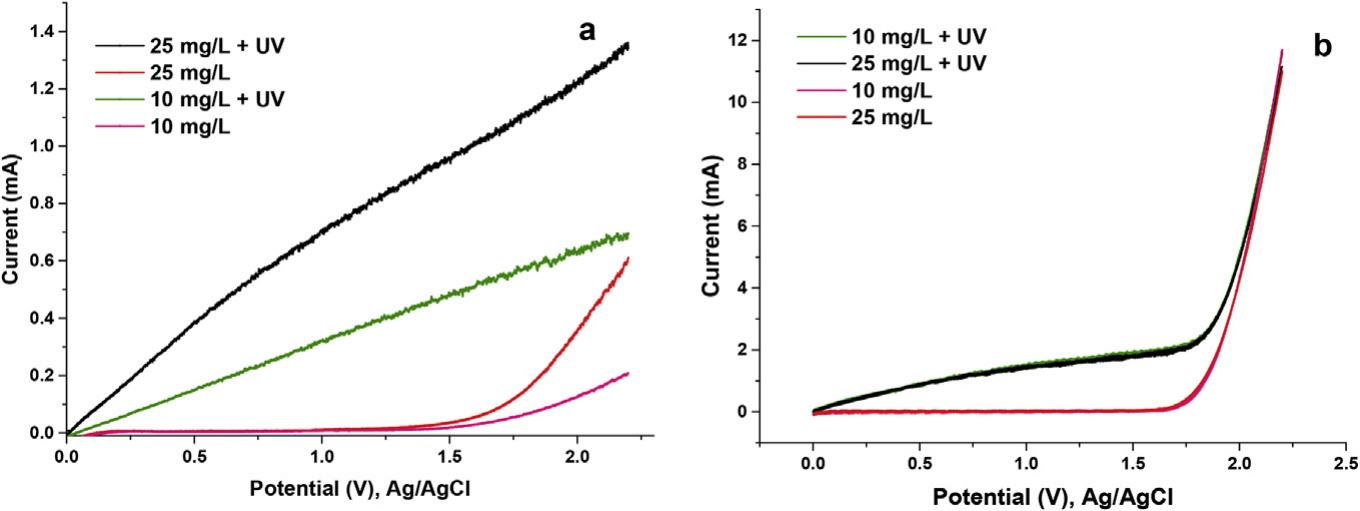
Linear scanning voltammetry of phenol solutions a) voltammetry in the absence of electrolyte. b) Voltammetry in presence of $0.1\;M\;Na_{2}SO_{4}$.

**Fig. 3:**
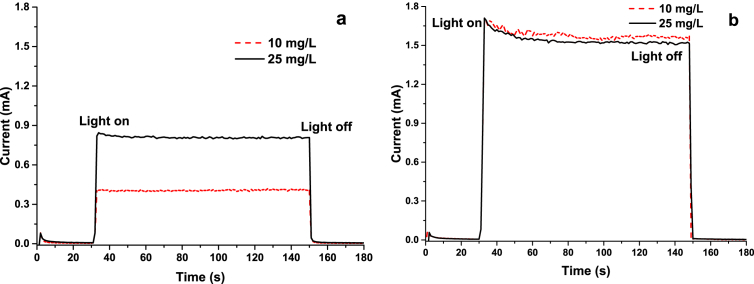
Chronoamperometry of phenol solutions a) Chronoamperometry in the absence of electrolyte. b) Chronoamperometry in presence of $\;0.1\;M\;Na_{2}SO_{4}$.

Fig. 2 and Fig. 3 show the voltammetry and chronoamperometry essays of the different concentrations of phenol in presence and absence of 0.1M of $Na_{2}SO_{4}$.

### 1.2 Degradation of phenol

Table 1 shows the degradation percentage of phenol by the photoelectrochemical oxidation along with the conditions used.

**Table 1:** Phenol degradation percentage and operating conditions.

Test	pH	$C_{0}\;\left[mg/L\right]$ Initial concentration of phenol	$\phi_{a}$ [V] Anodic potential	Degradation (%)	Q [C/L] Specific charge	$J_{avg}$$\left(\frac{mA}{cm^{2}}\right)$ Average current density
						
1	5	10	0.80	57.14	131.7	0.088
2	5	25	0.80	34.95	116.3	0.078
3	5	10	1.60	73.76	214.5	0.143
4	5	25	1.60	33.11	159.7	0.106
5	9	10	0.80	31.12	125.9	0.084
6	9	25	0.80	20.72	104.5	0.070
7	9	10	1.60	53.94	212.7	0.142
8	9	25	1.60	23.25	198.6	0.132
9	3.64	17.5	1.20	74.58	154.7	0.103
10	7	17.5	0.53	21.44	92.5	0.062
11	7	4.89	1.20	72.95	185.5	0.124
12	7	17.5	1.20	31.06	155.0	0.103
13	7	30.1	1.20	24.55	164.9	0.110
14	7	17.5	1.87	42.35	210.6	0.140
15	10.36	17.5	1.20	29.08	164.8	0.110

With all the data obtained, the analysis of variance shown in Table 2 was made to identify the influence of the variables in the elimination of the pollutant.

**Table 2:** Analysis of variance for the photoelectrochemical degradation of phenol.

Source	Sum of Squares	Df	Mean Square	F-Ratio	P-Value
A: pH	1570.13	1	1570.13	56.86	0.0006
B: Concentration	2514.85	1	2514.85	91.07	0.0002
C: Potential	415.095	1	415.095	15.03	0.0117
AA	238.106	1	238.106	8.62	0.0324
AB	59.1328	1	59.1328	2.14	0.2033
AC	13.9656	1	13.9656	0.51	0.5088
BB	162.516	1	162.516	5.88	0.0597
BC	187.695	1	187.695	6.80	0.0478
CC	3.73075	1	3.73075	0.14	0.7283
Total error	138.079	5	27.6158	–	–
Total (corr.)	5462.06	14	–	–	–

**Fig. 4:**
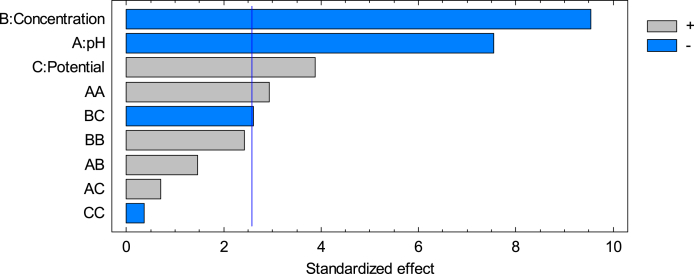
Standardized Pareto chart for degradation.

The Pareto chart in Fig. 4 evidence the importance of the variables in the elimination of the pollutant.

**Fig. 5:**
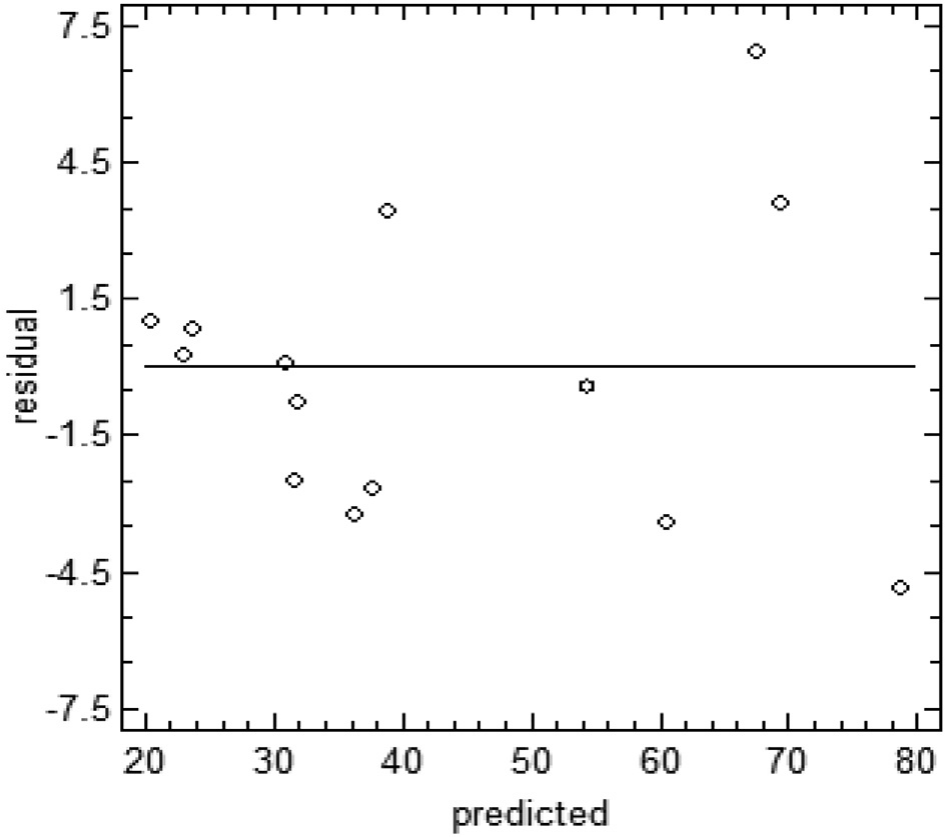
Residual graph for the photoelectrochemical oxidation of phenol.

The residual graph in Fig. 5 shows the change in variability according to the fluctuations of the predicted value of pollutant degradation by the regression model.

**Fig. 6:**
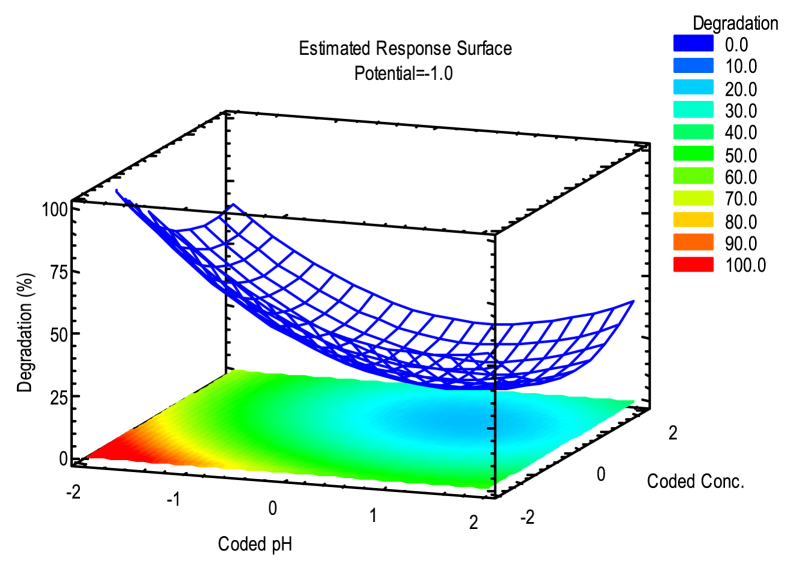
Estimated Surface response for the photoelectrochemical degradation of phenol.

In the surface response shown in Fig. 6, the optimal conditions to achieve the highest degradation percentage of the contaminant are observed.

### 1.3 Kinetics of photolysis, photocatalysis, and electrochemical oxidation in the degradation of phenol

**Fig. 7:**
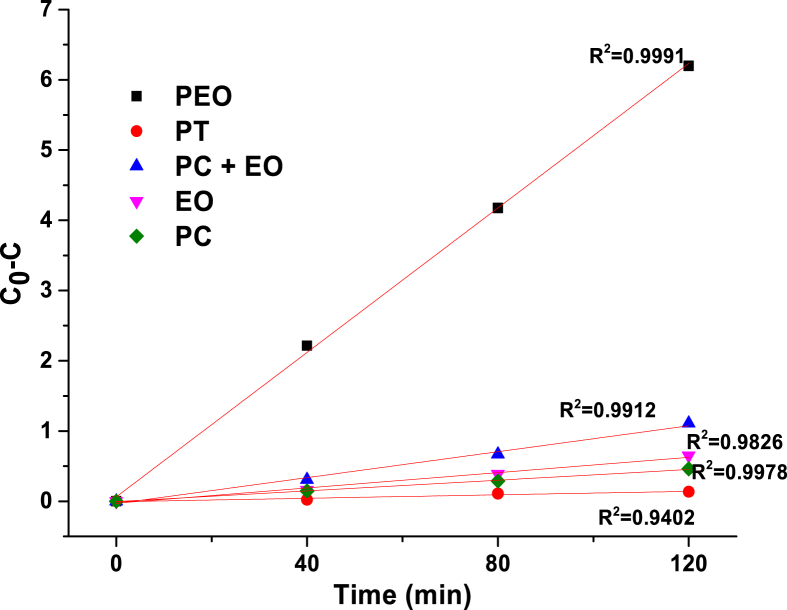
Adjustment of phenol degradation kinetics for photolysis (PT), photocatalysis (PC), electrochemical oxidation (EO), Photocatalysis + electrochemical oxidation (PC + EO) and photoelectrochemical oxidation (PEC).

The phenol degradation by Photolysis (PT), Photocatalysis (PC), Electrochemical oxidation (EO), Photocatalysis + Electrochemical oxidation (PC + EO) by separate and photoelectrochemical oxidation (PEC) well fit a zero-order kinetics. The adjustments were plotted in Fig. 7 and the zero rate constants for all the treatments are shown in Table 3.

**Table 3:** Rate constants of phenol degradation by the different processes and correlation coefficients.

Treatment	Rate constant $\left(mM\times min^{-1}\right)$	$R^{2}$	Phenol degradation (%)
PEC	$5.525\times 10^{-4}$	0.9991	73.76
PC + EO	$9.350\times 10^{-5}$	0.9912	11.60
EO	$5.440\times 10^{-5}$	0.9826	6.79
PC	$3.995\times 10^{-5}$	0.9978	4.81
PT	$1.232\times 10^{-5}$	0.9402	1.42

### 1.4 Electrical energy per order (EEO)

Table 4 shows the specific energy consumption by separate of the different technologies that are integrated into the photoelectrochemical oxidation technique.

**Table 4:** Energy consumption of the evaluated processes.

Treatment	Cell current (mA)	Cell potential (V)	Nominal LED´s potential (kW)	EEO (kWh/L)
Photoelectrochemical Oxidation	1.70	1.84	$5.72\times 10^{-3}$	599.8
Photocatalysis + Anodic Oxidation	0.09	1.60	$5.72\times 10^{-3}$	11923.8
Anodic Oxidation	0.09	1.60	–	8367.7
Photocatalysis	–	–	$5.72\times 10^{-3}$	3732.6
Photolysis	–	–	$5.72\times 10^{-3}$	32075.6

## 2 Experimental design, materials and methods

### 2.1 Experimental procedure

**Fig. 8:**
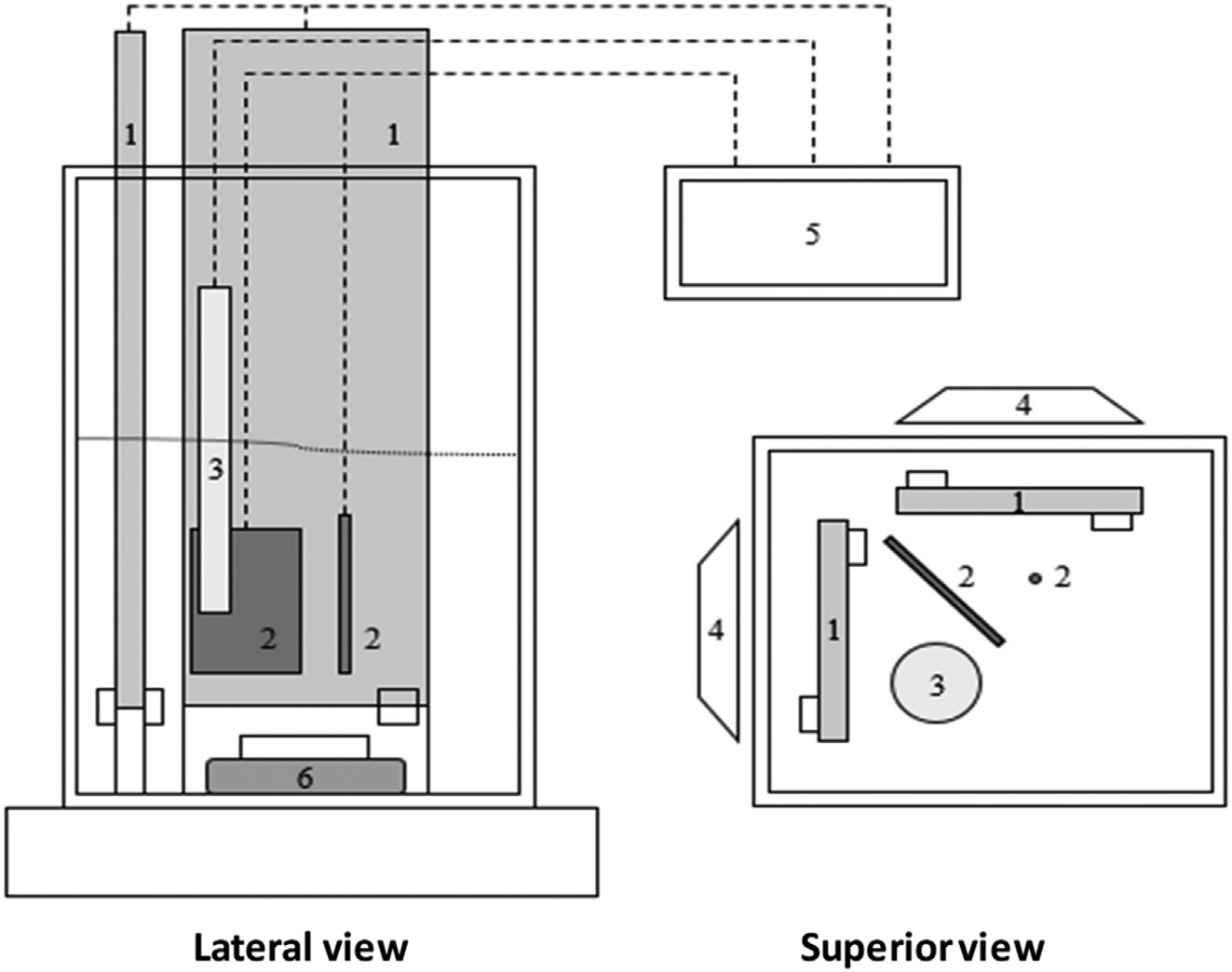
Scheme of the electrochemical cell: (1) Working electrode$\;\mathrm{Ti}O_{2}$, (2) Counter electrodes (Pt). (3) Reference electrode, (4) light source, (5) Potentiostat/galvanostat, (6) stirring bar.

The electrochemical cell was elaborated in acrylic, considering the dimensions of the light source and the electrodes. Those were fixed in the cell without affecting its active area (back (SE) illumination), which allowed carrying out experiments under stable conditions in terms of the geometric variables that may affect the mass transfer in the process. The contaminant solution was stirred during the tests with the aim to decrease the limitations associated with the diffusion phenomena. The scheme can be appreciated in Fig. 8.

All the solutions were prepared with deionized water type II prior to the experiments. An aliquot of the contaminant solution was taken and diluted in the cell until reaching the initial phenol concentration needed. Three values of pH were chosen for the tests (pH 5, 7 and 9) to evaluate the behavior of the Photoelectrochemical process in all the range of the pH scale. The initial pH of the solution was adjusted with 0.5N of NaOH or $H_{2}SO_{4}$ when it was needed. The LED's were turned on 15 minutes previous to the experiments to stabilize the emission of photons, then the LED's power was verified to assure the same amount of radiation in all the tests.

The work potentials were supplied by means of the potentiostat/galvanostat shown in Table 6, Table 7 and Fig. 8. For each test, 0.1M of $Na_{2}SO_{4}$ was used as supporting electrolyte, 60 ml of solution were prepared for the treatment, and samples of 1.5 ml were taken each 40 minutes during 2 hours. Later, they were diluted 6.66 times (1.5 ml:10 ml) using deionized water type II and the concentration of phenol was determined by the direct photometric method using 4-aminoantipyrine, the absorbance measurements were made at 510 nm as it is described in the ASTM D1783standard method [1].

**Table 6:** List of equipment and instruments.

Equipment (Model)	Brand
Analytical Balance (AS 220/C/2)	Radwag
Ag/AgCl Reference electrode (930–15)	Gamry
UVA LEDs 3W 25 mW/cm2	LED World
Digital multiparameter (Sension + MM150)	Hach
heating and stirring plate (PC-420D	Corning
Potentiostat/Galvanostat (series G 750)	Gamry
Radiometer (HD 2102.2)	Delta OHM
Probe, 315 nm–400 nm, (LP 471 UVA)	Delta OHM
UV–Vis Spectrophotometer UV1800	Shimadzu

**Table 7:** Operation levels of the experimental design.

Initial pH		$C_{0}\;\left(mg/L\right)$		$\phi_{a}$ (V)	
Low level (−1)	High level (+1)	Low level (−1)	High level (+1)	Low level (−1)	High level (+1)
5	9	10	25	0.80	1.60

The operating variables that were fixed for the development of the experimental design were: supporting electrolyte concentration$\;\left(0.1\;M\;Na_{2}SO_{4}\right)$, reaction volume(60ml), stirring velocity(600rpm), temperature (28±2), reaction time (2hours). The degradation percentage was the response variable, which it is calculated as follows.

(1) $$\%\;phenol\;degradation=\frac{C_{0}-C}{C_{0}}\times 100\;$$

Where $C_{o}$ is the initial concentration of phenol and C is the final concentration of the pollutant.

A photolysis test (without a working potential and photo-anodes), an electrochemical oxidation test (in the absence of light), a photocatalysis test (without working potential) and a photocatalysis + anodic oxidation by separate, were also carried out with the aim to obtain the degradation percentages of phenol in each one of the techniques mentioned. The data well fitted a zero-order kinetic that is described in the equation (2).

(2) $$C_{0}-C=kt\;$$

This was made with the aim to determine if there is a synergistic effect of the different processes in the photoelectrochemical oxidation of phenol.

The voltammetric tests were carried out with potential barriers from 0 to 2.2 V (50mV/s) and the chronoamperometries at a potential of1.2V. Through these techniques, the effect of the supporting electrolyte and the concentration of phenol on the current generated in the system in the absence and presence of light was elucidated. All the measurements reported take as reference the Ag/AgCl electrode and were made at pH 5.

### 2.2 Reagents and equipment

Table 5 and Table 6 show all the reagents, materials and equipment used to carry out this work. All the chemical compounds were used as received without further purification. NOMAD (Canadian research group) donated the $FTO/TiO_{2}-nanoparticles$ electrodes employed. The elaboration methodology is described in detail in [2]. The active area of each electrode was 2.5cm×2.5cm (thickness 3 mm) and the measurements of the platinum electrode used were 1.1cm×1.65cm (sheet) and 1.8cm×0.1cm (wire).

**Table 5:** Reagents and materials used in the tests.

Material (Purity)	Brand	Application
Sulfuric acid (95–98%)	Fisher Scientific	Tests
Deionized water type II	–	Tests
FTO/TiO_2_-np electrodes	Fabricated	Tests
Platinum electrodes	Fabricated	Tests
Sodium chloride (≥99.8%)	Sigma - Aldrich	Tests
Phenol crystals	Carlo Erba	Tests
Sodium Hydroxide (98.3%)	Agenquímicos	Tests
Sodium sulfate anhydrous (99.8%)	Agenquímicos	Tests
4 – Aminoantipyrin (98%)	Panreac	Direct photometric method
Ammonium chloride (99.9%)	Fisher Scientific	Direct photometric method
Potassium hexacyanoferrate (98%)	Panreac	Direct photometric method
Ammonium hydroxide (28.89%)	Fisher Scientific	Direct photometric method

### 2.3 Experimental design

Through a central rotary composite design with uniform precision at two levels, the data for the photoelectrochemical oxidation of phenol was obtained. All the tests were in function of the initial pH, the initial concentration of contaminant and potential applied. In the development of this work, 20 experimental runs were made (8 factorial points, 6 axial points and 1 central point replicated by 5 times). The tests were carried out in a random order with the aim to avoid systematic errors. For the analysis of the data, some statistics software such as Statgraphics Centurion XV and Minitab 17 were used. Table 7 summarizes the operating levels of the experimental design.

### 2.4 Electrical energy per order (EEO)

With the aim to calculate the energy consumption of the treatment evaluated, the figure of merit proposed by the international union of pure and applied chemistry (IUPAC) was used. The equation that best fits a zero-order kinetics in advanced oxidation processes is presented as follows:

(3) $$\text{EEO}=\frac{1000\;\text{* P*t}}{\text{V*log}\left({\text{C}}_{\text{i}}/{\text{C}}_{\text{f}}\right)}$$

Where P is the power source that is supplied to the system (kWh), t is the total time of treatment(h), V is the total volume of reaction (L) and $C_{0}$ and C are the initial and final concentrations of the contaminant respectively (M) [3].

## References

[1] ASTM (1995). Standard Test Methods for Phenolic Compounds in Water 1. Test.

[2] Xu F., Benavides J., Ma X., Cloutier S.G. (2012). Interconnected TiO_2_ nanowire networks for PbS quantum dot solar cell applications. J. Nanotechnol..

[3] Bolton J.R., Bircher K.G., Tumas W., Tolman C.A. (2001). Figures-of-merit for the technical development and application of advanced oxidation technologies for both electric- and solar-driven systems (IUPAC Technical Report). Pure Appl. Chem..

