# Supplementary material for: Experimental data on the photoelectrochemical oxidation of phenol: Analysis of pH, potential and initial concentration
Source: Data Brief. 2019 Apr 25;24:103949. doi: 10.1016/j.dib.2019.103949 (PMC6503135; doi:10.1016/j.dib.2019.103949)
Supplement: Multimedia component 1 [file mmc1.docx]

Santiago de Cali, 30/01/2019

Data in Brief

# Editors

Dear Editors

The authors of the paper entitled: **Experimental data on the photoelectrochemical oxidation of phenol: Analysis of pH, potential and initial concentration.** by Villota Zuleta Julieth Andrea , Rodríguez Acosta John Wilman, Castilla Acevedo Samir Fernando, Marriaga Cabrales Nilson de Jesús, Fiderman Machuca-Martínez do not have conflicts of interest for the publication of this work

Yours sincerely

Prof. Fiderman Machuca Martinez

Corresponding author
